# Eco-Friendly Approach for the Construction of Superhydrophobic Coating on Stainless Steel Metal Based on Biological Metal–Organic Framework and Its Corrosion Resistance Performance

**DOI:** 10.3390/ma16134728

**Published:** 2023-06-30

**Authors:** Rasmiah Saad Almufarij, Howida Abouel Fetouh El Sayed, Mohamed Elshahat Mohamed

**Affiliations:** 1 Department of Chemistry, College of Science, Princess Nourah Bint Abdulrahman University, P.O. Box 84428, Riyadh 11671, Saudi Arabia; rsmufrrig@pnu.edu.sa; 2 Chemistry Department, Faculty of Science, Alexandria University, Alexandria 21568, Alexandria Governorate, Egypt; howida_fetouh@yahoo.com; 3 Faculty of Advanced Basic Sciences, Alamein International University, Alamein City 51718, Matrouh Governorate, Egypt

**Keywords:** superhydrophobic coating, stainless steel, metal–organic framework, corrosion resistance, polarization, impedance

## Abstract

In this paper, we present a sustainable approach for the creation of superhydrophobic (SP) coating on a stainless-steel substrate based on a biological metal–organic framework (MOF). The MOF was synthesized using aspartic acid as a linker and copper ions as a core metal. Two SP coatings were well constructed on stainless steel utilizing electrodeposition of nickel (Ni) and nickel altered by MOF (Ni@Bio-MOF) coatings followed by soaking in a solution of stearic acid in ethanol. The results of Fourier transform infrared spectroscopy demonstrate that the stearic acid-grafted nickel coating (Ni@SA) and the stearic acid-grafted Ni@Bio-MOF composite (Ni@Bio-MOF@SA), were effectively deposited on the stainless steel. The wettability findings displayed that the water contact angle of Ni@SA and Ni@Cu-As MOF@SA are 160° ± 1.1°, and 168° ± 1.2°, respectively. The prepared SP coating was also found to be chemically and mechanically stable. The results show that the Ni@SA coating maintains SP characteristics in a pH range of 3–11 while the Ni@Cu-As MOF@SA coating retained SP characteristics in a pH range of 1–13. Additionally, the superhydrophobic Ni@SA coating demonstrated SP characteristics up to a length of abrasion equal to 1300 mm, while the Ni@Cu-As MOF@SA coating exhibited SP characteristics up to a length of abrasion equal to 2700 mm. Furthermore, the Ni@SA and Ni@Cu-As MOF@SA coatings exhibited significantly improved corrosion protection in a 0.5 M NaCl solution compared with bare stainless steel, with protection efficiencies of approximately 94% and 99%, respectively. The results of this study demonstrate that the proposed approach is a promising method for the fabrication of eco-friendly and corrosion-resistant SP coatings on stainless steel substrate.

## 1. Introduction

Extremely non-wettable surfaces, also known as superhydrophobic (SP) surfaces are surfaces that have a contact angle greater than 150 degrees and are highly resistant to water [1]. These surfaces have attracted interest due to their potential applications in various fields such as antifouling technologies, microfluidic devices, biomedical, solar cells, sensors, drag reduction, oil–water separation, and corrosion resistance [2,3,4,5]. However, creating SP surfaces can be difficult and most methods require extreme conditions, especially when environmental issues are present. There are a variety of techniques used to create SP surfaces, including electrodeposition, spraying, anodization, electrospinning, sol-gel, and chemical vapor deposition [6,7]. Electrodeposition is a technique that is relatively simple, low-cost, and flexible, making it an excellent option for creating artificial SP surfaces [8]. The scalability of the electrodeposition method depends on a number of factors such as the size and shape of the object being coated. While it may be feasible to use electrodeposition to coat large objects, such as wings, aircrafts, or the rotor blades of wind turbines, doing so would require significant modifications to the electrodeposition system to ensure uniform coating and adhesion over such large surfaces.

By raising surface roughness, a vital prerequisite for superhydrophobicity and lowering surface energy, another crucial prerequisite for superhydrophobicity, SP films with significant water repellency can be produced [9]. As roughness increases, the surface can trap air pockets within the gaps between surface features, preventing the liquid from fully wetting the surface and resulting in a Cassie–Baxter state and superhydrophobicity. The degree of superhydrophobicity also depends on the shape of the protrusions and their spacing [10].

Historically, perfluorinated compounds have been used to lower surface energy because of their own very low surface energy, but these compounds have been shown to have toxic and harmful environmental effects [11]. As a result, eco-friendly techniques, and materials for producing SP surfaces are required. To enhance roughness, researchers have utilized a range of nanomaterials such as metal–organic frameworks (MOFs), carbon nanotubes, SiO_2_, TiO_2_, ZnO, and CuO [12,13,14,15,16,17]. MOFs are combination materials with porous structures and periodic network arrangements produced via organic linkers and core metal ions. These have gained popularity due to their unique properties and potential applications [1]. However, traditional methods for creating MOFs have their own drawbacks, while electrochemical techniques offer various benefits, such as clean-up, convenience of use, mild reaction conditions and metal ions formed in situ via anodic oxidation, which avoids the need to utilize problematic anions [18]. Recently, biological metal–organic frameworks, Bio-MOFs, have been gaining reputation as renewable framework materials that are recyclable, and green [19]. Bio-MOFs use biomolecules, such as amino acids, peptides, nucleobases, sugars, and other bio-based materials, as building blocks and water as a solvent and have been used to produce SP surfaces [19].

Stainless steel (SS) is widely used in the marine engineering, aerospace, automotive, petrochemical, nuclear engineering and biomedical sectors [20,21]. Though SS has the advantages of an improved corrosion resistance, a good toughness and a high strength, it is subjected to severe corrosion in media containing chloride ions [22,23]. Corrosion is a significant problem for society as it affects both public safety and the economy [24,25,26]. The impact of corrosion on public safety can be significant as it can weaken the structural integrity of various infrastructure, such as buildings, bridges, pipelines, power plants, transportation vehicles, etc. Corrosion can potentially lead to failures and accidents that may cause injuries or fatalities and can also have serious consequences for the environment. Nuclear power plants are particularly susceptible to corrosion-related issues that can compromise their safety and reliability. Therefore, it is crucial to develop effective corrosion protection and mitigation strategies to ensure the safety and reliability of our infrastructure and systems. One of the most effective ways to reduce SS corrosion is by creating SP coats that drastically increase the corrosion resistance of SS [27]. Nickel is a valuable metal in the industrial sector, known for features such as corrosion resistance, hardness, and magnetism. The corrosion of SS is slowed when nickel is applied to it. When combined with superhydrophobicity, the nickel coat is able to deliver additional advantages, such as better corrosion resistance and self-cleaning properties [28].

The goal of this research is to create an SP coat on an SS substrate using Bio-MOF. The electrochemical process was utilized to prepare a Cu-As MOF coating which contains aspartic acid as the linker and copper as the core metal. Two rough coats of Ni, and Ni modified with Bio-MOF (Ni@Bio-MOF), were grafted on an SS substrate through electrostatic deposition. The coats were soaked in an ethanol solution containing stearic acid (SA) to create SP surfaces. To increase the mechanical and chemical stability of the SP coats on stainless steel, we innovatively employed a composite of Cu-As bio-MOF, synthesized by electrodeposition, and nickel. Stearic acid was utilized as a low-surface energy compound due to it being an eco-friendly substance that is more cost-effective in comparison with other options, such as toxic fluorinated polymers and silanes. The prepared SP coatings’ wettability, mechanical and chemical stability, and corrosion resistance in a solution of 0.5 M NaCl were evaluated.

## 2. Materials and Methods

### 2.1. Materials

The used substrate in our work is composed of smooth and flat sheets of 304 SS with dimensions of 2.0 cm × 2.0 cm × 0.1 cm. The chemical composition of the SS (wt%) is C (0.07%), Mn (1.50%), P (0.01%), Si (0.5%), Cr (18%), Ni (8.5%), and balanced Fe. Anhydrous ethanol, sulfuric acid, sodium hydroxide, boric acid, aspartic acid, stearic acid, nickel hexahydrate, and nickel sulphate were of the analytical-grade chemicals employed in the experiment.

### 2.2. The Construction of Cu-As MOF

The process of creating a copper aspartic acid metal–organic framework (Cu-As MOF) involved polishing a copper sheet using 200 and 1200 grit sandpaper to eliminate the oxide layer on its surface, followed by a rinsing of it with distilled water and ethanol. An electrochemical bath made up of 100 mL aspartic acid at a concentration of 4.5 gL^−1^ was used, with the copper sheet serving as both the anode and cathode. A direct current was applied at a current density of 0.05 A/cm^2^ for 1 h (an investigation into the effects of varying current densities over different time intervals was conducted, and the reported current density value and time in this study represents the optimal condition determined through our experimentation) at a temperature of 55 ± 1.5 °C [1]. As the process continued, a precipitate of blue color is deposited at the anode. The precipitate was then scratched and dried at room temperature. The MOF was further dried at 100 °C in an oven for 2 h to yield the final product, the Cu-As MOF.

### 2.3. Superhydrophobic Coating Manufacture

The SS was prepared for electrodeposition by first polishing it with sandpaper of different grades, starting with a rough grade (200) and gradually moving to a smoother grade (1200). The SS was then cleaned by immersing it in the soap for 10 min, rinsing it with distilled water, and submerging it in 2.0 M H_2_SO_4_ for 1 min. After this preparation, an area of 2.0 cm × 1.0 cm of SS was immersed in the bath for electrodeposition. The parameters for this process, which involved coating the SS with Ni and a modified form of Ni by Bio-MOF (Ni@Bio-MOF), are listed in Table 1. The used parameters in the table (concentrations of NiCl_2_.6H_2_O, NiSO_4,_ and H_3_BO_3_ as well as the time and potential of deposition) were based on previous studies [27]. Different quantities of Bio-MOF (0.1, 0.2, 0.3, 0.4 and 0.5 gL^−1^) were studied and the reported quantity is the optimum which gives the higher superhydrophobicity. The SS acted as the cathode and was parted from the platinum anode of the same area by 2.0 cm. Once the coatings were applied, the distilled water was used to clean the coated substrates and left to air dry overnight. The coatings were then modified by submersion in an ethanolic solution of 0.01 M SA for 15 min, washing them with ethanol, and allowing them to dry for 24 h. The coated SS with Ni modified with SA (Ni@SA) and the coated SS with Ni@ Bio-MOF modified with SA (Ni@Cu-As MOF@SA) were subjected to various characterizations processes.

### 2.4. Surface Characterization

Thermogravimetric analysis was utilized to assess the thermal stability of the Cu-As MOF (TGA-Shimadzu-50, Shimadzu Corp., Kyoto, Japan). With the aid of a Bruker Tensor 37 FTIR Fourier transform infrared spectrophotometer (Bruker, Singapore), the surfaces’ chemical compositions were investigated. The morphology of the surface of the SP coatings was inspected using an SEM (JSM-200 IT, JEOL, Tokyo, Japan) via optical contact angle goniometer (model 190-F2) and 5 µL water droplet, the water contact angle (CA) and water sliding angle (SA) were calculated. The CA and SA readings that were presented were calculated as the average of three tests made at various points on the substrate.

### 2.5. Chemical Stability

The SP films were put into different solutions with pH levels ranging from 1.0 to 13 and were left there for 1 h. The CA and SA were measured after each pH change. To alter the pH of the solution, sulfuric acid and sodium hydroxide were utilized. The chemical stability results are based on an average of two tests undertaken on different substrates.

### 2.6. Mechanical Abrasion

To evaluate the mechanical properties of the SP films, two experiments were conducted: a sand impact test and an abrasion test. In the abrasion test, the SP film was applied to an 800 grade SiC paper and a pressure of 5.0 kPa was applied to it. The CA and SA of a water droplet were measured for every 100 mm of abrasion. In the sand impact experiment, 50 g of sand was dropped from a height of 60 cm onto SP-coated SS. The CAs and SAs were measured for every 50 g of sand that hit the SP surface to determine the material’s water superhydrophobicity. To make a further assessment of the mechanical properties of the SP coatings, CAs and SAs were measured after every 50 g of sand was dropped onto the SP surface. The existing data are an average of two tests taken on different substrates.

### 2.7. Corrosion Test

With a three-electrode cell and a Pt sheet serving as the auxiliary electrode and an Ag/AgCl electrode serving as the reference electrode, the electrochemical tests were conducted using an ACM frequency response analyzer. The used SS samples, working electrode, were bare and SP-coated SS with Ni@SA and Ni@Cu-As MOF@SA films, which were then coated with an epoxy layer except for a 1 cm^2^ area was left bare for the test solution. Prior to performing electrochemical tests, the SS samples were submerged in a 0.5 M NaCl solution at room conditions for 30 min to establish a rest potential. The electrochemical impedance spectroscopy tests used a frequency range between 0.01 and 1.0 × 10^4^. The potentiodynamic polarization tests were performed within a potential range of ±250 mV around the rest potential. The tests were repeated to ensure accuracy with an error margin of 2%.

## 3. Results and Discussion

### 3.1. Thermogravimetric Results of the Prepared Cu-As MOF

**Figure 1:**
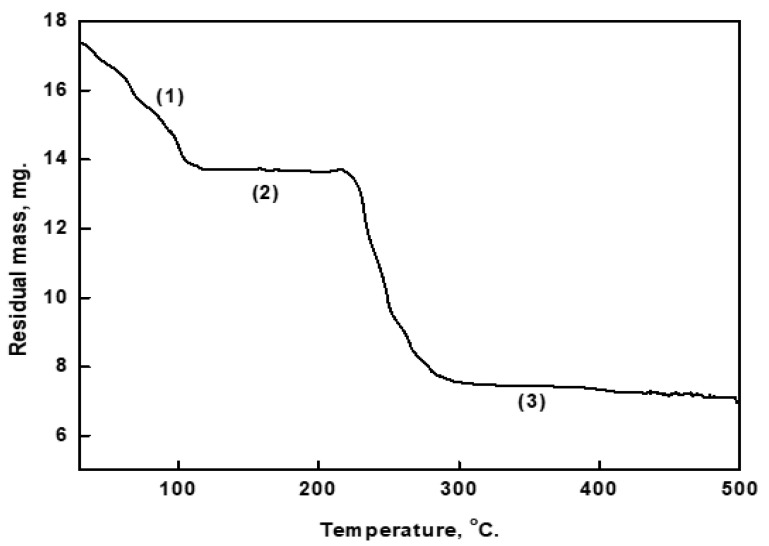
Thermogravimetric graph for Cu-As MOF in air atmosphere, (1) first region, (2) second region, and (3) third region.

The thermogravimetric results of Cu-aspartic acid MOF, shown in Figure 1, would likely center around the three distinct regions observed in the graph, and the changes in weight that occur in each region. The first region, between 32 and 101 °C, may be characterized by a relatively low rate of weight loss as the MOF loses adsorbed water or other weakly bound species. The second region, between 101 and 218 °C, may be characterized by a more rapid weight loss as the MOF loses more strongly bound species or undergoes structural changes. The third region, between 218 to 288 °C, may be characterized by a slower rate of weight loss, as the MOF reaches its maximum decomposition temperature. This region may indicate that the MOF is losing its structural integrity.

### 3.2. FTIR Results

**Figure 2:**
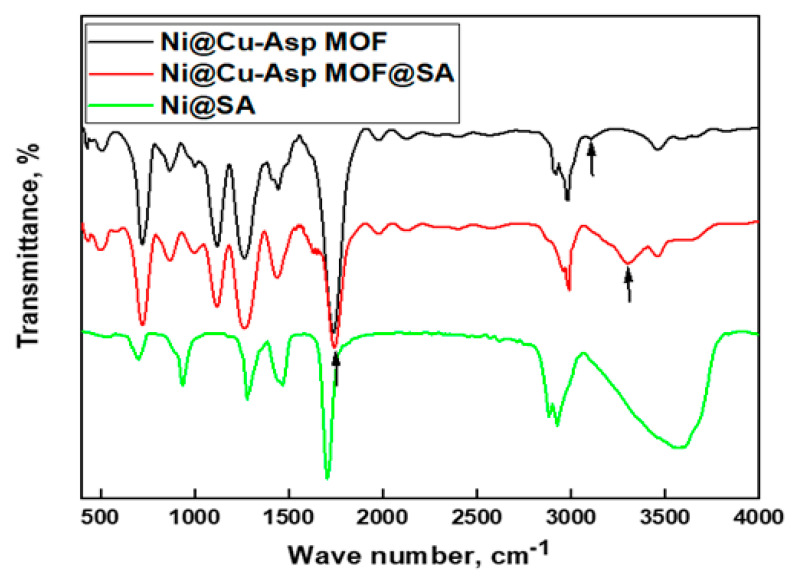
FTIR spectra for superhydrophobic stainless steel coated by Ni@Cu-As MOF, Ni@Cu-As MOF@SA, and Ni@SA.

The FTIR spectra of coated SS with Ni@Cu-As MOF, Ni@Cu-As MOF@SA, and Ni@SA are presented in Figure 2. The FTIR results for the coated SS with Ni@Cu-As MOF likely indicate the presence of several functional groups in the material. The band at 3463 cm^−1^, and 3111 cm^−1^ may be due to the N-H_2_ stretch of aspartic acid [27]. The bands at 2980 cm^−1^, and 2899 cm^−1^ may be due to the presence of C-H symmetry and a symmetry vibration of -CH_2_- groups [1]. The band at 1724 cm^−1^ is due to the stretching vibration of C=O and the band at 1439 cm^−1^ may be due to the presence of C-N stretching vibrations, indicating the presence of amine groups [1]. The band at 864 cm^−1^ is due to the stretching of C-H bonds in an aspartic acid compound [1]. The band at 728 cm^−1^ is due to the presence of Ni(OH)_2_ bending in the coating while the band at 506 cm^−1^ is due to the metal–oxygen stretching vibrations and the band at 429 cm^−1^ is due to the metal–oxygen bending vibrations, indicating the coordination of the copper ions with the oxygen atoms of the carboxylate and amine groups [1].

The spectrum of the SS coated with Ni@Cu-As MOF@SA displays similar bands to that of the Cu-As MOF, but with slight changes in the position of the band of the C=O stretch and the N-H_2_ stretch band, which appear at 1733 cm^−1^ and 3294 cm^−1^, respectively. This suggests that the Cu-As MOF has been doped with SA [27].

The spectrum of the coated SS with Ni@SA coat displays a band at 3530 cm^−1^ which is likely due to the presence of the hydroxyl groups of stearic acid [27]. The bands at 2932 cm^−1^, and 2894 cm^−1^ may be due to the presence of C-H symmetry and a symmetry vibration of -CH_2_- groups [27]. The band at 1698 cm^−1^ is associated with the stretching vibrations of C=O in the stearic acid and the band at 1454 cm^−1^ is likely due to the bending vibrations of the CH_2_ groups in the stearic acid [29]. The bands at 1255 and 971 cm^−1^ are due to CH stretch. The band at 689 cm^−1^ is likely attributed to the presence of Ni(OH)_2_ bending in the coating [30].

### 3.3. SEM and Wettability

**Figure 3:**
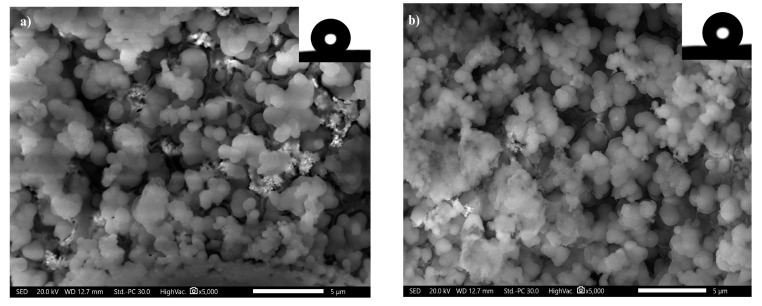
SEM of superhydrophobic coated stainless steel with (**a**) Ni@SA and (**b**) Ni@Cu-As MOF@SA. The image of water droplets on the superhydrophobic coats is inserted as an inset.

The SEM of the SS coated by Ni@Cu-As MOF@SA, and Ni@SA are presented in Figure 3. The discussion of SEM results of SP-coated SS with Ni@Cu-As MOF@SA, and Ni@SA likely centers around the differences in microstructure and surface roughness between the two coatings. The SEM micrographs likely show that the coating made with Ni@Cu-As MOF@SA has smaller circular microstructures compared with the coating made with Ni@SA. The usage of an MOF in the Ni@Cu-As MOF@SA coating may operate as a nucleation center for the electrodeposition process, speeding up the nucleation process rather than crystal growth and producing smaller structures, increasing the surface’s roughness. The smaller size of the microstructures may also contribute to the SP properties of the coating, as smaller structures can lead to a more roughness, and stable water-repellent coating. The wettability of the Ni@SA, and Ni@Cu-As MOF@SA was examined by the measurement of CA. The Ni@SA has CA of 160° ± 1.1°, and an SA of 4° ± 0.1°, while Ni@Cu-As MOF@SA has a CA of 168° ± 1.2°, and an SA of 1° ± 0.1°, so the two coats showed excellent SP properties. The micrograph of the water droplet on the SP prepared with Ni@SA and Ni@Cu-As MOF@SA is shown as an inset in Figure 3. The rolling/bouncing of the water droplet on the SS coated by Ni@Cu-As MOF@SA is illustrated in Video S1.

### 3.4. Chemical Stability

**Figure 4:**
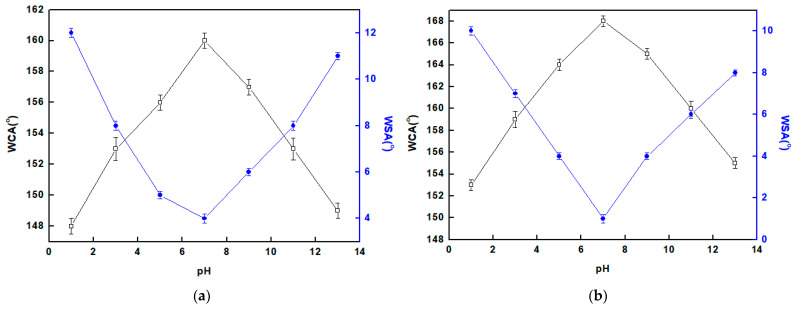
The change of solution pH with the CA and SA of the superhydrophobic coated stainless steel by (**a**) Ni@SA, and (**b**) Ni@Cu-As MOF@SA.

**Figure 5:**
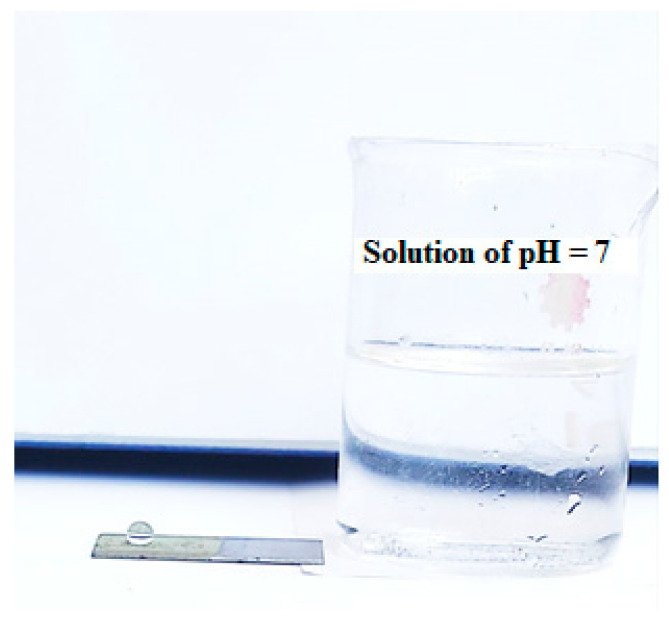
The shape of water droplet on the coated stainless steel by Ni@Cu-As MOF@SA film after 5 hours immersion in a solution of pH 7.

Chemical stability is considered an essential requirement for SP coatings to work well over time in harsh solution conditions. The correlations between the CAs and SAs of water droplets on the SP coatings and the solution pH are depicted in Figure 4. The shape of the water droplet on the SS coated with Ni@Cu-As MOF@SA after being immersed in a solution of pH 7 for 5 h is illustrated in Figure 5. Video S2 demonstrates the superhydrophobicity and rolling of a water droplet on the SS coated with a Ni@Cu-As MOF@SA surface after immersion in the pH 7 solution for 5 h. According to the findings, Ni@SA films are SP in the pH range of 3–11, while Ni@Cu-As MOF@SA films are SP in the pH range of 1–13, where the CAs are frequently higher than 150° and the SAs are less than 10°. The chemical stability of SP-coated SS with Ni@Cu-As MOF@SA is higher than that of SS coated with Ni@SA because the Cu-As MOF enhances the coating superhydrophobicity and provides an additional layer of protection. The SP-coated SS with Ni@Cu-As MOF@SA has a superior chemical stability to numerous values that have been reported previously [31,32,33,34].

### 3.5. Mechanical Stability

**Figure 6:**
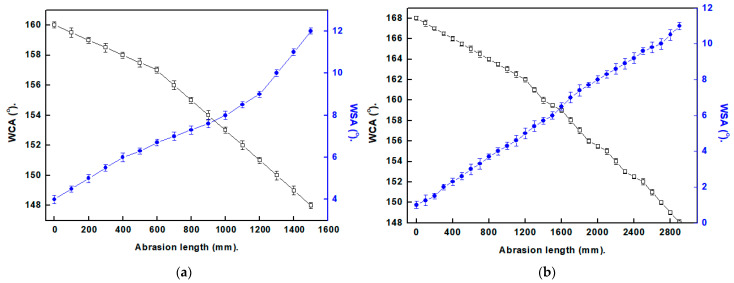
The alteration in CAs and SAs with the length of abrasion for superhydrophobic coated stainless steel with (**a**) Ni@SA, and (**b**) Ni@Cu-As MOF@SA.

SP surfaces often have limited practical applications due to their mechanical fragility. When touched with a finger, some surfaces with SP characteristics can crash [4]. The produced SP films’ resistances to mechanical abrasion were assessed utilizing abrasion and sand impact tests. Figure 6 depicts the variations in CAs and SAs of the manufactured SP films with respect to the abrasion length. The Ni@SA SP film maintains its SP characteristics up to a 1300 mm abrasion length. In comparison, the SP Ni@Cu-As MOF@SA film preserves its SP characteristics up to a 2700 mm abrasion length. The SP-coated SS with Ni@Cu-As MOF@SA exhibits larger abrasion resistance than numerous stated values [27,35]. The mechanical abrasion test for SS coated with Ni@Cu-As MOF@SA film for abrasion length of 20 cm is shown in Video S3.

The mechanical abrasion resistance of SP-coated SS via Ni@Cu-As MOF@SA is higher than that of SP SS coated with Ni@SA only because the MOF layer enhances the superhydrophobicity and provides an additional layer of protection, resulting in a more durable and abrasion-resistant coating [36,37]. 

**Figure 7:**
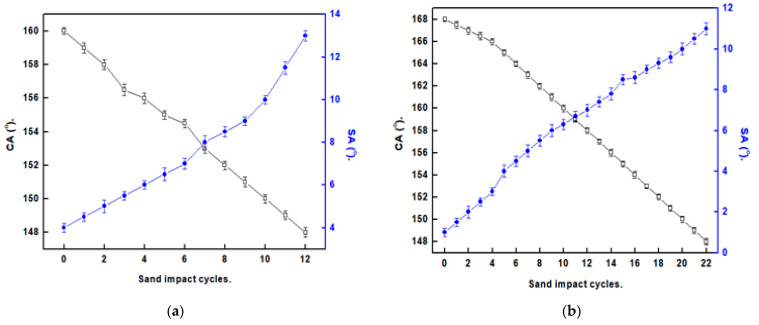
The relation between the sand impact cycles and the CAs and SAs of superhydrophobic-coated stainless steel with (**a**) Ni@SA, and (**b**) Ni@Cu-As MOF@SA.

As seen in Figure 7, the sand abrasion assessments were undertaken to evaluate the mechanical performance of the SP coatings. The Ni@SA film maintains SP characteristics up to 10 sand impact cycles, while the Ni@Cu-As MOF@SA film exhibits superhydrophobicity up to 20 sand impact cycles. Ni@Cu-As MOF@SA exhibit a sand impact resistance larger than several previously stated values [33,38].

### 3.6. Corrosion Measurements

#### 3.6.1. Potentiodynamic Polarization Results

**Figure 8:**
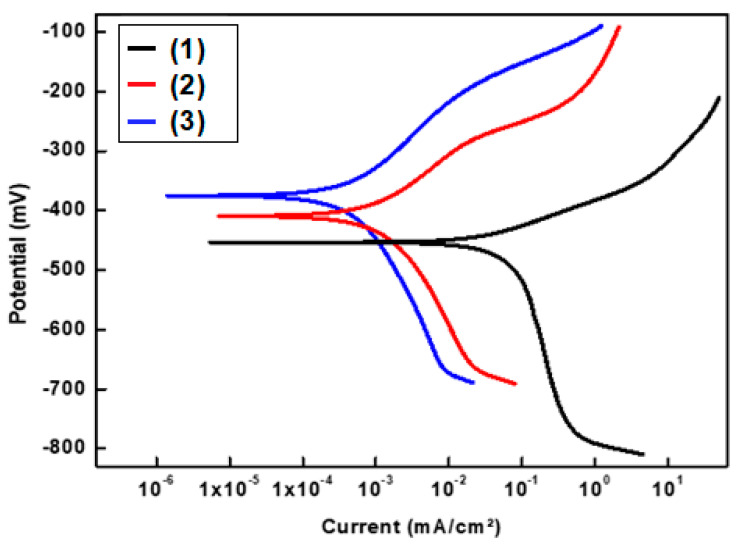
The potentiodynamic polarization plots of (1) bare stainless steel, and superhydrophobic-coated stainless steel by (2) Ni@SA, and (3) Ni@Cu-As MOF@SA in 0.5 M NaCl solution.

The corrosion behaviors of uncoated and SP-coated SS by Ni@SA, Ni@Cu-As MOF@SA were studied using the potentiodynamic polarization technique. The potentiodynamic polarization plots of bare and SP-coated SS in 0.5 M NaCl are shown in Figure 8. The observation of limited diffusion currents during cathodic polarization suggests that the cathodic process is governed by the transfer of oxygen gas from the bulk to the electrode surface. Pitting corrosion for bare SS or the development of a passive layer for SS cured with an SP coating prevent the formation of an ideal anodic Tafel area [39,40]. 

Table 2 shows the potentiodynamic polarization variables for bare and SP-coated SS, containing corrosion potential (E_corr_), protection efficiency (%P), and corrosion current density (i_corr_). The %P was determined utilizing Equation (2) [41].

%P = [(i_o_ − i_)/_i_o_] × 100 (1)

where, i_o_ and i are the corrosion current densities of the bare SS and the SP-coated SS, respectively. The i_corr_ value for coated SS with Ni@SA is lower than that for bare SS due to the superhydrophobicity of the coated SS. Air trapped in the SP coating microstructures can diminish the surface area between the solution and SS, which causes the i_corr_ value to fall more quickly [42]. The doping of the superhydrophobic Ni@SA coat with MOF enhances the SP property, leading to a greater decline in the contact area between the medium and SS. Therefore, SS coated with Ni@Cu-As MOF@SA has a higher protection efficiency than SS coated with Ni@SA.

#### 3.6.2. Electrochemical Impedance Spectroscopy Results

**Figure 9:**
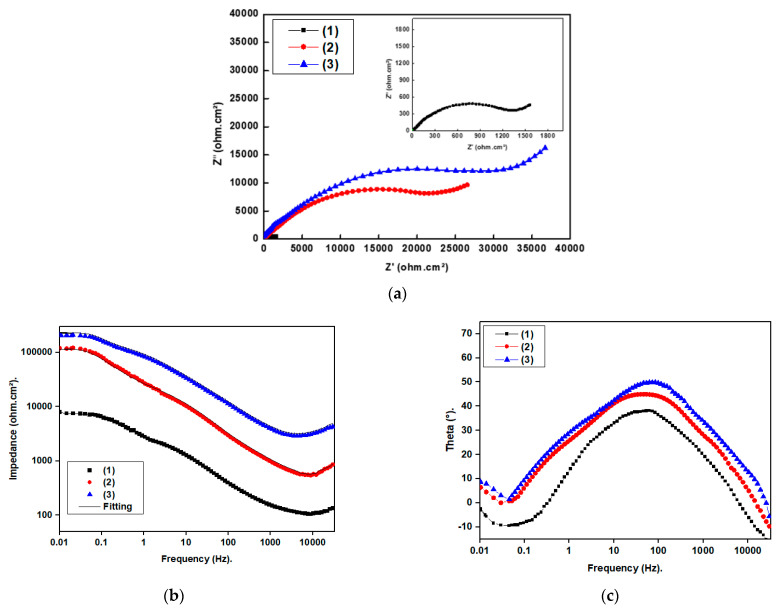
(**a**) Nyquist, (**b**) Bode, and (**c**) Theta plots of (1) bare stainless steel, and superhydrophobic coated stainless steel by (2) Ni@SA and (3) Ni@Cu-As MOF@SA in 0.5 M NaCl.

Nyquist, Bode and Theta plots of the bare and SP-coated SS in a 0.5 M NaCl solution are presented in Figure 9. The Nyquist plots, shown in Figure 9a, exhibit a diffusion tail at low frequency and a depressed capacitive semicircle at high frequency. The depressed capacitive semicircle observed at high frequencies in the Nyquist plots is due to the interfacial charge transfer reaction [43]. The diffusion tails observed at low frequencies are attributed to mass transfer. Based on these observations, it can be inferred that the improved charge transfer resistance of SS coated with Ni@SA compared with bare SS is attributed to the presence of a protective SP layer. The SS coated with Ni@Cu-Asp MOF@SA exhibits the largest capacitive semicircle, suggesting that it provides the highest level of protection. The incorporation of MOF onto Ni@SA enhances the superhydrophobicity of the surface, making the superhydrophobic Ni@Cu-Asp MOF@SA coating more effective in restricting the diffusion of corrosive species such as Cl^−^ and H_2_O into the SS substrate.

When the superhydrophobic-coated SS was immersed in a 0.5 M NaCl solution, it demonstrated significantly higher impedance magnitudes at lower frequencies on the Bode plots, shown in Figure 9b, compared with bare steel. This clearly indicates that the created SP coatings have successfully protected the SS substrate. The phase angle plot, shown in Figure 9c, shows two time constants at low and intermediate frequencies. The time constant observed at the low-frequency region is attributed to the unprotective corrosion products of bare SS or the protective SP coating. On the other hand, the time constant observed at the moderate frequency is attributed to the electrical double layer. The theta angle, which is approximately 45 degrees at a moderate frequency, indicates that the corrosion process is under diffusion control.

**Figure 10:**
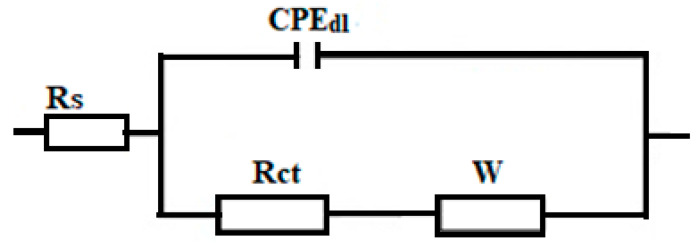
The equivalent circuit model.

The equivalent circuit displayed in Figure 10 was utilized to fit the results of the electrochemical impedance spectroscopy experiment and the Zsimpwin program was utilized to calculate the impedance parameters. The components of the equivalent circuit include the solution resistance (R_s_), the charge transfer resistance (R_ct_), the double-layer constant phase element (CPE_dl_), and the Warburg element (W). Table 3 demonstrates the electrochemical impedance spectroscopy parameters for both bare SS and SP-coated SS. Equation (2) was utilized to estimate the %P [27]:

%P = [(R_ct_ − R_cto_)/R_ct_] × 100 (2)

where the charge transfer resistances for uncoated and SP-coated SS are R_cto_ and R_ct_, respectively. Table 3 presents the obtained impedance parameters. It is clear that, each of R_ct_ and %P of the bare SS < SS + Ni @SA < SS + Ni@Cu-As MOF@SA. The corrosion performance of the SP-coated SS by Ni@Cu-As MOF@SA is greater than many previously documented values [44,45,46,47] and lower than other previously reported values [48,49].

The corrosion resistance of SP-coated SS with a Ni@Cu-As MOF@SA coating is higher than that of SS coated with Ni@SA. This may be due to the way in which the Cu-As MOF increases the superhydrophobicity and forms a protective barrier against corrosive agents such as water and oxygen, thus slowing down the corrosion process and enhancing the corrosion resistance of the coating.

## 4. Conclusions

In our study, an eco-friendly approach to the construction of SP coatings on SS metal based on biological metal–organic frameworks (MOFs) was developed. The MOF was synthesized using aspartic acid as the organic linker and copper ions as the metal center. The water contact angles of SS coated with Ni@SA and Ni@Cu-As MOF@SA are 160° ± 1.1°, and 168° ± 1.2°, respectively. 

The chemical stability results show that Ni @SA coating maintains SP characteristics in the pH range of 3–11, whereas the Ni@Cu-As MOF@SA coating retains SP characteristics in the pH range of 1–13. The mechanical stability results show that the created SP Ni@SA coating demonstrates SP characteristics up to an abrasion length of 1300 mm, while the Ni@Cu-As MOF@SA coating exhibits SP characteristics up to an abrasion length of 2700 mm. The corrosion resistance of the coated SS was also significantly improved. The corrosion current density of the coated SS with Ni@SA is 0.0041915 µA/cm^2^, and Ni@Cu-As MOF@SA is 0.0041915 µA/cm^2^, which was much lower than that of the bare SS (0.0710569 µA/cm^2^). The results of this study demonstrate that the proposed approach is a promising method for the construction of eco-friendly SP coatings with excellent corrosion resistance.

## Figures and Tables

**Table 1:** Different parameters for electrodeposition of Ni@SA and Ni@Cu-As MOF@SA coating on the SS surface.

Factor	Ni@SA	Ni@Cu-As MOF@SA
NiCl_2_.6H_2_O	40 gL^−1^	40 gL^−1^
NiSO_4_	176 gL^−1^	176 gL^−1^
H_3_BO_3_	60 gL^−1^	60 gL^−1^
Bio-MOF	0.0 gL^−1^	0.4 gL^−1^
Time of electrodeposition	6.0 min	6.0 min
Deposition potential	11.0 Volt	11.0 Volt

**Table 2:** The potentiodynamic polarization parameters for the bare and the superhydrophobic coated stainless steel in 0.5 M NaCl solution.

Deposit	-E_corr_ mV	β_a_ mV/Decade	-β_c_ mV/Decade	i_corr_ µA/cm^2^	%P
Bare stainless steel	455.03	44.323	467.05	0.0710569	---
stainless steel + Ni@SA	434.56	95.915	211.46	0.0041915	94.1
stainless steel + Ni@Cu-As MOF@SA	392.42	112.02	181.69	0.0007017	99.0

**Table 3:** The electrochemical impedance spectroscopy parameters for the bare and superhydrophobic-coated stainless steel in 0.5 M NaCl solution.

Coat	Rs (Ohm.cm^2^)	n_1_	CPE_dl_ × 10^−6^ (s^n^ Ω^−1^ cm^2^)	W × 10^−4^	R_ct_ (Ohm.cm^2^)	%P
Bare stainless steel	2.1	0.77	311.1	444.4	1312	---
Stainless steel + Ni@SA	3.9	0.74	56.2	22.3	28,420	95.3
Stainless steel + Ni@Cu-As MOF@SA	4.4	0.72	35.3	18.9	47,512	97.2

## Data Availability

All data in this study are available on request.

## Supplementary Materials

The following supporting information can be downloaded at: https://www.mdpi.com/article/10.3390/ma16134728/s1, Video S1. The rolling of a water droplet on the superhydrophobic coated stainless steel by Ni@Cu-Asp MOF@SA film. Video S2. The superhydrophobicity and rolling of a water droplet on the coated stainless steel by Ni@Cu-Asp MOF@SA film after immersion in pH 7 solution for 5 h. Video S3. The mechanical abrasion test for SS coated with Ni@Cu-Asp MOF@SA film for abrasion length of 20 cm.
